# Sex differences in the associations of dietary protein intake with lean mass and grip strength in children and adolescents

**DOI:** 10.1080/15502783.2025.2471471

**Published:** 2025-02-24

**Authors:** Yanfei Wang, Dan Sun, Zhongxin Zhu

**Affiliations:** a Xiaoshan Affiliated Hospital of Wenzhou Medical University, Hospital Tendering Management Center, The First People’s Hospital of Xiaoshan District, Hangzhou, China; b Community Health Service Center of Guali, Department of Pediatrics, Hangzhou, Zhejiang, China; c Xiaoshan Affiliated Hospital of Wenzhou Medical University, Department of Osteoporosis Care and Control, The First People’s Hospital of Xiaoshan District, Hangzhou, Zhejiang, China

**Keywords:** Dietary protein, musculoskeletal health, adolescent nutrition, NHANES

## Abstract

**Background:**

Childhood and adolescence are critical developmental periods during which dietary protein plays a crucial role in musculoskeletal health. While the significance of protein in muscle development is well acknowledged, the complex associations between dietary protein intake and musculoskeletal parameters during these stages remain incompletely elucidated.

**Methods:**

This cross-sectional study utilized data from the National Health and Nutrition Examination Survey (NHANES) 2011–2014 cycles, including 3,455 children and adolescents aged 8–19 years. Dietary protein intake was assessed through two 24-hour dietary recalls. Appendicular lean mass index (ALMI) and combined grip strength were measured as indicators of musculoskeletal health. Multivariate linear regression models and smooth curve fitting techniques were employed to analyze associations.

**Results:**

Higher protein intake was positively associated with both ALMI (β = 0.003, 95% CI: 0.002, 0.004, *p* < 0.001) and combined grip strength (β = 0.043, 95% CI: 0.027, 0.058, *p* < 0.001) in fully adjusted models. Notably, sex-specific effects were observed, with stronger associations in boys, particularly in the 8–11 years age group.

**Conclusions:**

This study reveals significant positive associations between dietary protein intake and musculoskeletal health indicators in children and adolescents, with pronounced sex-specific effects. These findings underscore the importance of adequate protein intake during critical developmental periods and may inform targeted nutritional strategies for optimizing long-term musculoskeletal health.

## 1. Introduction

Childhood and adolescence constitute a pivotal developmental window characterized by remarkable physiological plasticity, where optimal protein intake critically shapes metabolic programming and long-term nutritional trajectories [1,2]. Dietary protein plays a fundamental role in this physiological equilibrium, simultaneously stimulating muscle protein synthesis and attenuating proteolysis, thereby promoting the maintenance and accretion of lean body mass over time [3]. The ontogeny of lean mass follows a distinct triphasic trajectory – rapid accretion during growth, relative stability through young- and middle-adulthood, followed by a gradual decline in later life – with peak muscular development during the second and third decades serving as a critical indicator of long-term musculoskeletal health potential [4,5].

Recent years have witnessed a paradigm shift in nutritional epidemiology, with heightened focus on the musculoskeletal fitness of pediatric and adolescent populations [6,7]. This emerging emphasis is particularly significant, given the profound implications for longitudinal health outcomes extending into adulthood. While adequate protein is essential for growth and development, nuanced nutritional strategies are crucial; excessive protein consumption can precipitate negative health consequences, including obesity and metabolic dysregulation in later life [8,9]. Within this complex context, optimal protein consumption is hypothesized to be fundamental for precise myogenesis, comprehensive neuromuscular development, and sustained functional capacity [10,11].

Muscle mass and strength emerge as pivotal indicators of overall musculoskeletal health and physical performance [12,13]. An expanding corpus of evidence, encompassing diverse populations and age demographics, consistently demonstrates a robust positive association between dietary protein intake and both lean mass and muscle strength [14–16]. Although the anabolic effects of protein on muscle hypertrophy and function are well-established in adult populations [17,18], its specific developmental impact during childhood and adolescence remains incompletely characterized. This critical knowledge gap is particularly significant, given the transformative potential of early nutritional interventions to modulate lifelong health trajectories and optimize muscular development. Our study aims to elucidate the intricate associations between dietary protein intake and two fundamental musculoskeletal parameters: appendicular lean mass index (ALMI) and grip strength – a reliably measured proxy for muscle function and development [19] —within a diverse population of children and adolescents.

## 2. Methods

### 2.1. Study design and population

We leveraged data from the National Health and Nutrition Examination Survey (NHANES), a comprehensive, cross-sectional survey of the United States population. NHANES provides publicly accessible, de-identified data, serving as a cornerstone for numerous epidemiological investigations. All NHANES protocols were approved by the National Center for Health Statistics’ ethics review board, with written informed consent procured from participants’ parents or legal guardians.

Data from two consecutive NHANES cycles (2011–2014) were amalgamated, initially encompassing 4,450 children and adolescents aged 8–19 years. Following a rigorous exclusion process, we eliminated individuals with incomplete dietary data (*n* = 606), ALMI measurements (*n* = 327), and combined grip strength assessments (*n* = 62). The resulting analytical data comprised 3,455 subjects, ensuring robust statistical power for subsequent analyses.

### 2.2. Exposure and outcome variables

The primary exposure variable, dietary protein intake, was quantified through two 24-hour dietary recalls conducted by trained interviewers. The initial interview was performed in-person at the Mobile Examination Center, with a follow-up interview via telephone or mail within 3–10 days, enhancing data reliability. Nutrient values were assigned using the US Department of Agriculture Food and Nutrient Database for Diet Studies [20]. The average total protein intake from these two recalls served as our exposure metric.

Our primary outcome variables, ALMI and combined grip strength, were selected as key indicators of musculoskeletal health. ALMI was calculated as appendicular lean mass [kg] divided by height squared [m^2^], derived from dual-energy X-ray absorptiometry (DXA) scans. These scans, performed in the supine position using a Hologic QDR-4500A fan-beam densitometer (Hologic, Inc., Bedford, MA), provided precise measurements of appendicular lean mass, defined as the sum of lean tissue in the arms and legs. Height measurements adhered to standardized protocols in the Mobile Examination Center.

Grip strength was evaluated using a Takei Digital gripper force gauge (model T.K.K.5401). This instrument measured the maximum force exerted by hands in kilograms. The assessment protocol was rigorously standardized: the dynamometer was adjusted to participants’ hand size, participants were instructed to exert maximum force, and the test was repeated thrice for each hand with a 60-second inter-trial rest period. Measurements were deemed valid when participants maintained proper posture and a 90° angle with the index finger on the dynamometer handle. Our analysis utilized the combined hands’ grip strength, representing the sum of the largest reading from each hand, thus providing a comprehensive measure of upper body strength.

### 2.3. Confounding variables

Drawing from extant literature, we identified and collected data on key potential confounders: age (stratified into 8–11, 12–15, and 16–19 years), sex, race/ethnicity (categorized as non-Hispanic White, non-Hispanic Black, Mexican American, and other race/ethnicity), ratio of family income to poverty, body mass index (BMI), and vitamin D and calcium intakes. This comprehensive set of confounders allowed for robust adjustment in our statistical models.

The ratio of family income to poverty, a socioeconomic indicator computed by the National Center for Health Statistics, ranges from 0 to 5 and is based on thresholds established by the US Census Bureau. BMI, calculated as body weight (kg) divided by the square of height (m^2^), served as a measure of body composition. Vitamin D and calcium intakes, crucial for musculoskeletal health, were derived from the average of two individual 24-hour dietary recall interviews, ensuring alignment with our protein intake assessment methodology.

### 2.4. Statistical analyses

Our statistical approach was structured into three interconnected steps, designed to provide a comprehensive analysis of the relationships between dietary protein intake and musculoskeletal health indicators:

1. Baseline characterization: We stratified the subjects by dietary protein intake quartiles and presented the distribution of baseline characteristics. Continuous variables were expressed as mean ± standard deviation, while categorical variables were presented as percentages. Inter-group differences were assessed using χ^2^ tests for categorical variables, one-way ANOVA for normally distributed continuous variables, and Kruskal-Wallis H tests for skewed distributions, ensuring appropriate statistical treatment of each variable type.
2. Multivariate analysis: We employed a series of multivariate linear regression models to evaluate associations between dietary protein intake and both ALMI and grip strength. Adhering to the STROBE statement recommendations [21]: we constructed three models: Model 1 (unadjusted), Model 2 (adjusted for age, sex, and race), and Model 3 (fully adjusted for all screened covariates). This stepwise approach allowed us to assess the impact of confounding factors on the observed associations. Additionally, we conducted subgroup analyses using stratified linear regression models to explore potential effect modifications. Interaction terms between subgroup indicators were tested, followed by likelihood ratio tests, to rigorously assess the significance of any observed effect modifications.
3. Non-linear relationship exploration: Recognizing the potential for complex relationships, we employed smooth curve fitting techniques and generalized additive models to explore and confirm potential non-linear associations between dietary protein intake and both ALMI and grip strength. This approach allowed for the detection of nuanced relationships that might not be captured by linear models alone.

All statistical analyses were conducted using R software (version 3.4.3) and EmpowerStats (X&Y Solutions, Inc., Boston, MA). Two-sided *p* values less than 0.05 were considered statistically significant.

## 3. Results

The characteristics of study population based on dietary protein intake quartiles are presented in Table 1. Higher protein consumption correlated with increased age and male predominance, with 71.9% boys in the highest quartile compared to 34.0% in the lowest. Vitamin D and calcium intake showed positive associations with protein consumption. Notably, ALMI and combined grip strength increased across protein intake quartiles. Socioeconomic factors, including race/ethnicity and family income to poverty ratio, differed across quartiles.

**Table 1.** Characteristics of study population based on dietary protein intake quartiles.

Protein intake (g/d)	Q1 (≤52.845)	Q2 (52.865–68.265)	Q3 (68.27–87.605)	Q4 (≥87.62)	P value
Age (years)	13.1 ± 3.3	12.5 ± 3.4	13.0 ± 3.4	14.1 ± 3.4	<.001
Sex (%)					<.001
Boy	34.0	44.3	53.6	71.9	
Girl	66.0	55.7	46.4	28.1	
Race/Ethnicity (%)					<.001
Non-Hispanic White	22.9	27.6	26.4	24.4	
Non-Hispanic Black	32.1	28.2	25.6	23.4	
Mexican American	17.8	22.1	21.6	24.0	
Other race/ethnicity	27.2	22.1	26.4	28.2	
Moderate activities (%)					<.001
Yes	31.5	25.8	29.6	36.0	
No	32.8	27.0	28.5	35.2	
Unrecorded	35.8	47.2	41.9	28.8	
Ratio of family income to poverty	1.9 ± 1.5	2.0 ± 1.6	2.0 ± 1.6	2.1 ± 1.6	.022
Body mass index (kg/m^2^)	22.7 ± 6.3	22.2 ± 6.5	22.2 ± 5.9	22.6 ± 5.6	.190
Vitamin D intake (μg/d)	5.3 ± 17.9	6.6 ± 6.1	8.0 ± 7.5	10.2 ± 10.4	<.001
Calcium intake (mg/d)	635.0 ± 280.6	911.1 ± 304.0	1095.3 ± 378.1	1443.2 ± 601.1	<.001
Appendicular lean mass index (kg/m^2^)	6.4 ± 1.6	6.4 ± 1.7	6.5 ± 1.5	7.1 ± 1.7	<.001
Combined grip strength (kg)	50.0 ± 18.3	48.6 ± 20.1	51.0 ± 19.8	61.2 ± 23.9	<.001

**Figure 1.**
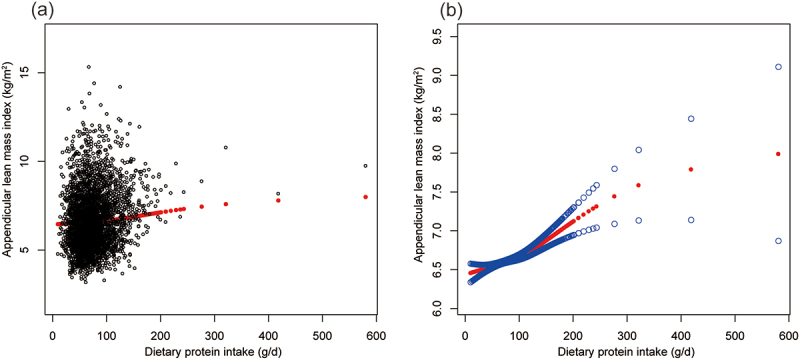
The association between dietary protein intake and appendicular lean mass index. (a) Each black point represents a sample. (b) Solid red line represents the smooth curve fit between variables. Blue bands represent the 95% of confidence interval from the fit.

**Figure 2.**
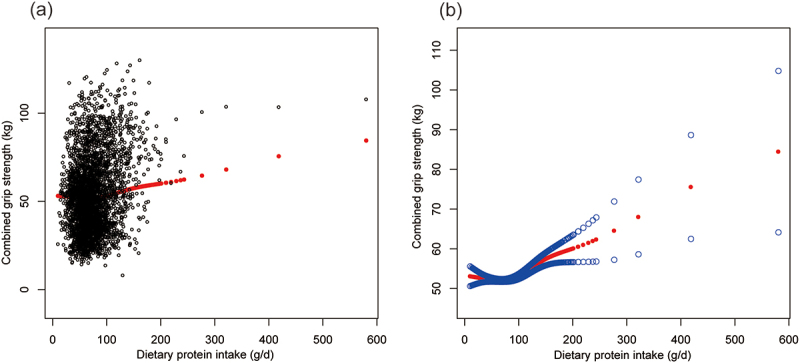
The association between dietary protein intake and combined grip strength. (a) Each black point represents a sample. (b) Solid red line represents the smooth curve fit between variables. Blue bands represent the 95% of confidence interval from the fit.

Multivariate regression analyses elucidated significant positive associations between protein intake and both ALMI and combined grip strength (Table 2). In the fully adjusted model, accounting for potential confounders, protein intake demonstrated a robust positive correlation with ALMI (β = 0.003, 95% CI: 0.002, 0.004, *p* < 0.001) and combined grip strength (β = 0.043, 95% CI: 0.027, 0.058, *p* < 0.001). Quartile analysis revealed a dose-response relationship for ALMI, with the highest protein intake quartile (Q4) exhibiting the strongest association compared to the lowest quartile (Q1). For combined grip strength, Q4 similarly showed a significant positive association. These positive and trend non-linear relationships were confirmed by Figures 1–2.

Age, sex, race, ratio of family income to poverty, moderate activities, body mass index, vitamin D intake and calcium intake were adjusted.

Age, sex, race, ratio of family income to poverty, moderate activities, body mass index, vitamin D intake and calcium intake were adjusted.

**Table 2.** Association of protein intake (g/d) with appendicular lean mass index (kg/m^2^) and combined grip strength (kg).

	Model 1 β (95% CI)	Model 2 β (95% CI)	Model 3 β (95% CI)
ALMI	0.009 (0.007, 0.011)***	0.002 (0.000, 0.003)*	0.003 (0.002, 0.004)***
Protein intak Q4			
Q1	Reference	Reference	Reference
Q2	0.016 (−0.136, 0.169)	0.108 (−0.009, 0.225)	0.104
Q3	0.133 (−0.019, 0.285)	0.043 (−0.075, 0.160)	0.077 (0.010, 0.145)
Q4	0.645 (0.493, 0.797)	0.114 (−0.008, 0.235)	0.182 (0.103, 0.260)
P for trend	<0.001	0.160	<0.001
Combined grip strength	0.164 (0.143, 0.186)***	0.044 (0.031, 0.057)***	0.043 (0.027, 0.058)***
Protein intak Q4			
Q1	Reference	Reference	Reference
Q2	−1.404 (−3.349, 0.540)	0.289 (−0.861, 1.439)	0.070 (−1.034, 1.175)
Q3	0.995 (−0.949, 2.939)	−0.445 (−1.601, 0.711)	−0.845 (−2.004, 0.314)
Q4	11.214 (9.270, 13.158)	2.475 (1.278, 3.672)	1.603 (0.261, 2.945)
P for trend	<0.001	<0.001	0.128

Model 1: no covariates were adjusted.

Model 2: age, sex and race were adjusted.

Model 3: age, sex, race, ratio of family income to poverty, moderate activities, body mass index, vitamin D intake and calcium intake were adjusted.

*p < 0.05, **p < 0.01, ***p < 0.001.

**Figure 3.**
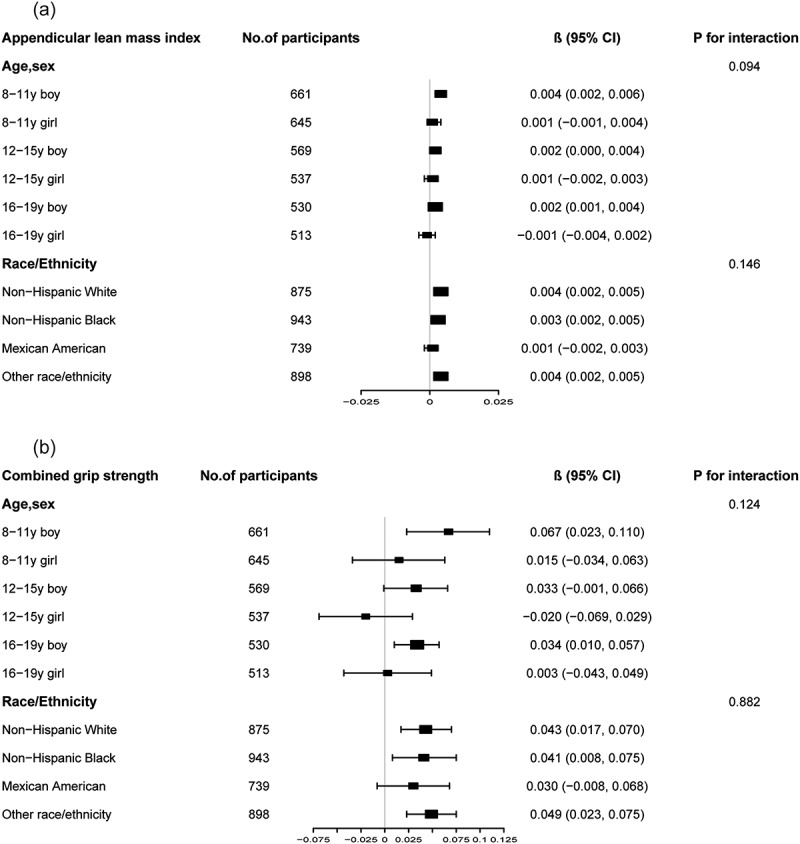
Subgroup analysis of the associations between dietary protein intake, appendicular lean mass index and combined grip strength.

Stratified analyses by age, sex, and race/ethnicity unveiled notable sex-specific effects. The positive correlations between dietary protein intake and ALMI were particularly pronounced in boys, especially in the 8–11 years age group (β = 0.004, 95% CI: 0.002, 0.006, *p* < 0.001), but were not statistically significant in girls (Figure 3a). A similar pattern was observed for grip strength, with significant positive correlations in boys, particularly in the 8–11 years age group (β = 0.004, 95% CI: 0.002, 0.006, *p* < 0.001), but not in girls (Figure 3b).

Age, sex, race, ratio of family income to poverty, moderate activities, body mass index, vitamin D intake and calcium intake were adjusted. In the subgroup analysis, the model is not adjusted for the stratification variable itself.

**Figure 4.**
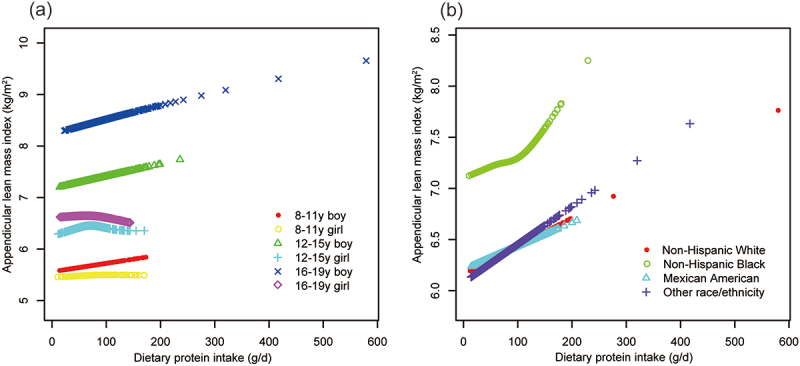
The association between dietary protein intake and appendicular lean mass index, stratified by age/sex, and race.

**Figure 5.**
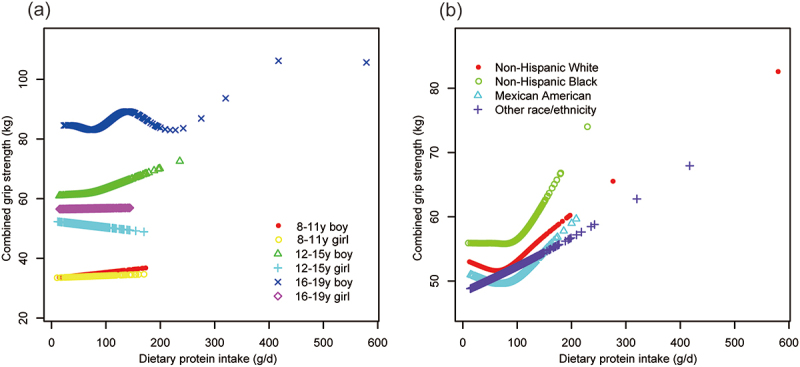
The association between dietary protein intake and combined grip strength, stratified by age/sex, and race.

Generalized additive models and smooth curve fittings (Figures 4–5) further substantiated these associations between dietary protein intake and both ALMI and grip strength. These models, stratified by age, sex, and race/ethnicity, revealed significant non-linear associations for both ALMI and grip strength, with varying patterns across subgroups.

Age, sex, race, ratio of family income to poverty, moderate activities, body mass index, vitamin D intake and calcium intake were adjusted. In the subgroup analysis, the model is not adjusted for the stratification variable itself.

Age, sex, race, ratio of family income to poverty, moderate activities, body mass index, vitamin D intake and calcium intake were adjusted. In the subgroup analysis, the model is not adjusted for the stratification variable itself.

## 4. Discussion

Our study unveils compelling associations between dietary protein intake and both ALMI and combined grip strength in children and adolescents, with notable sex-specific effects. These findings significantly advance our understanding of the critical role of protein intake during key developmental stages.

The relationship between dietary protein consumption and lean mass accrual in young individuals represents a complex and increasingly critical domain of nutritional science. Prospective studies have systematically demonstrated that elevated protein intake may substantially enhance skeletal muscle mass acquisition during adolescence [22], with longitudinal evidence establishing compelling links between higher total and morning protein consumption and increased skeletal muscle mass in late adolescence [23]. The physiological impact of protein intake on lean mass development is inherently nuanced, modulated by multifaceted factors including consumption timing [24,25], individual nutritional status [26], and obesity [27]. Current dietary recommendations for children and adolescents aged 8–19 years prescribe a protein intake of 0.75–1.05 g/kg body weight daily [28], yet comprehensive surveillance in Western developmental contexts conclusively reveals that actual protein consumption consistently exceeds these standardized recommendations by two- to three-fold [29]. Our findings revealed significant positive associations between dietary protein intake and musculoskeletal health indicators, with particularly pronounced effects observed in boys aged 8–11 years, which suggests that the existing recommendations may not fully account for the specific needs of different demographic groups. These results illuminate the critical need for more sophisticated, demographically stratified dietary guidelines that account for sex-specific and age-dependent variations in protein metabolism. By emphasizing the pivotal role of adequate protein intake during critical developmental periods, our study underscores the imperative for future nutritional strategies to incorporate more granular, quantitative assessments of protein intake’s physiological impacts, potentially revolutionizing our understanding of adolescent nutritional requirements.

Muscle weakness, rather than reduced muscle mass, has emerged as a more critical predictor of adverse outcomes in sarcopenia [30]. Recent large-scale studies, including one from the UK Biobank, have reported positive associations between protein intake and grip strength [31]. This finding is echoed in a Malaysian study demonstrating significant correlations between dietary protein intake and handgrip strength among adolescents [32], and further supported by research linking higher protein consumption with greater appendicular lean mass and handgrip strength in middle-aged adults [33]. However, the relationship between protein intake and grip strength exhibits variability across populations and age groups. While older adults showed a protective effect against grip strength loss with higher protein intake [34], this trend was not observed in younger individuals [35], suggesting an age-dependent effect.

It is important to recognize that excessive protein consumption in children and adolescents can lead to adverse effects, including an increased risk of obesity and metabolic diseases [36, 37], highlighting the necessity for balanced dietary approaches. Children have unique nutritional requirements relative to growth, necessitating adequate intake of energy and essential amino acids for optimal lean body mass deposition and normal development [38].

Our results underscore the importance of adequate protein intake for muscle mass and strength development in children and adolescents, with pronounced effects in boys. This aligns with a recent prospective cohort study demonstrating a significant positive longitudinal relationship between protein intake and muscle strength in males, but not in females [35]. These sex-specific differences may be influenced by various factors, including endocrine responses [39], body composition [40], and physical activity levels [41]. In both human and animal models, sex differences have been observed in hormonal and metabolic responses to dietary protein modulation. For instance, in mice, dietary protein dilution led to increased circulating fibroblast growth factor-21 (FGF21) in males but not females, indicating a sex-dependent response to dietary protein alterations [42]. This study also revealed that metabolic benefits associated with dietary protein dilution were absent in females, suggesting reduced sensitivity to these metabolic improvements. On the other hand, race/ethnicity and socioeconomic status represent critical determinants of dietary intake at cultural and social levels [43], with our study revealing significant disparities in protein consumption across racial/ethnic groups and family income levels. These nuanced findings elucidate the complex interplay between social stratification and nutritional patterns, thereby necessitating more precisely targeted and demographically informed nutritional interventions.

Our study leverages data from NHANES, a robust dataset that enhances the generalizability of our findings. Our focus on sex-specific effects and age-related variations provides valuable insights into the potentially differential impacts of protein intake across demographic subgroups. This stratified approach enhances the clinical relevance of our findings and may inform tailored nutritional strategies for specific populations. Despite the robust nature of our findings, several limitations warrant consideration. First, the cross-sectional design precludes the establishment of causal relationships between dietary protein intake and musculoskeletal parameters. Second, the reliance on 24-hour dietary recalls for protein intake estimation introduces potential recall bias and may not accurately reflect long-term dietary patterns. Although we employed the average of two nonconsecutive 24-hour recalls to mitigate day-to-day variability, this approach may still inadequately capture habitual protein consumption. Third, while our analyses adjusted for a wide range of covariates, residual confounding cannot be ruled out. Factors such as pubertal status and genetic predisposition to muscle development were not fully accounted for in our models. Fourth, while ALMI and grip strength are widely accepted measures of muscle mass and function, respectively, they do not capture the full spectrum of musculoskeletal health. Future studies should consider assessing different musculoskeletal components (strength, power, endurance) in various body regions (upper limbs, lower limbs, trunk) in children and adolescents.

## 5. Conclusion

Our findings elucidate a robust, positive association between dietary protein intake and musculoskeletal health indicators in children and adolescents, characterized by notable sex-specific effects. These results may contribute to the growing body of evidence linking early nutritional interventions to long-term health trajectories. Our findings have potential implications for developing targeted strategies aimed at preventing sarcopenia and related musculoskeletal conditions in later life, underscoring the importance of optimal protein intake during critical developmental periods.

## Data Availability

The data of this study are publicly available on the NHANES website (https://www.cdc.gov/nchs/nhanes/index.htm).

## References

[1] Maneschy I, Moreno LA, Ruperez AI, et al. Eating behavior associated with food intake in European adolescents participating in the HELENA study. Nutrients. 2022;14(15):3033. doi: 10.3390/nu14153033 35893887 PMC9332602

[2] Burd NA, McKenna CF, Salvador AF, et al. Dietary protein quantity, quality, and exercise are key to healthy living: a muscle-centric perspective across the lifespan. Front Nutr. 2019;6:83. doi: 10.3389/fnut.2019.00083 31245378 PMC6563776

[3] Atherton PJ, Smith K. Muscle protein synthesis in response to nutrition and exercise. J Physiol. 2012;590(5):1049–13. doi: 10.1113/jphysiol.2011.225003 22289911 PMC3381813

[4] Woo J. Sarcopenia. Clin Geriatr Med. 2017;33(3):305–314. doi: 10.1016/j.cger.2017.02.003 28689564

[5] McCarthy HD, Samani-Radia D, Jebb SA, et al. Skeletal muscle mass reference curves for children and adolescents. Pediatr Obes. 2014;9(4):249–259. doi: 10.1111/j.2047-6310.2013.00168.x 23776133

[6] Torres-Costoso A, Zymbal V, Janz KF, et al. Body composition and musculoskeletal fitness: a cluster analysis for the identification of risk phenotypes for pediatric sarcopenia. Clin Nutr. 2023;42(7):1151–1158. doi: 10.1016/j.clnu.2023.05.008 37244754

[7] Ooi PH, Thompson-Hodgetts S, Pritchard-Wiart L, et al. Pediatric sarcopenia: a paradigm in the overall definition of malnutrition in children? JPEN J Parenter Enteral Nutr. 2020;44(3):407–418. doi: 10.1002/jpen.1681 31328301

[8] Park YJ, Chung S, Hwang JT, et al. A review of recent evidence of dietary protein intake and health. Nutr Res Pract. 2022;16(Suppl 1):S37–s46. doi: 10.4162/nrp.2022.16.S1.S37 35651841 PMC9127511

[9] Arnesen EK, Thorisdottir B, Lamberg-Allardt C, et al. Protein intake in children and growth and risk of overweight or obesity: a systematic review and meta-analysis. Food Nutr Res. 2022;66:66. doi: 10.29219/fnr.v66.8242 PMC8861858 35261578

[10] Ganson KT, Nguyen L, Ali ARH, et al. “Eat more protein, build more muscle”: a grounded theory study of muscle-building behaviors among Canadian adolescents and young adults. Body Image. 2023;47:101635. doi: 10.1016/j.bodyim.2023.101635 37806066

[11] Hudson JL, Baum JI, Diaz EC, et al. Dietary protein requirements in children: methods for consideration. Nutrients. 2021;13(5):1554. doi: 10.3390/nu13051554 34063030 PMC8147948

[12] Cui H, Wang Z, Wu J, et al. Chinese expert consensus on prevention and intervention for elderly with sarcopenia (2023). Aging Med. 2023;6(2):104–115. doi: 10.1002/agm2.12245 PMC10242264 37287669

[13] Kirk B, Cawthon PM, Arai H, et al. The conceptual definition of sarcopenia: delphi consensus from the global leadership initiative in sarcopenia (GLIS). Age Ageing. 2024;53(3). doi: 10.1093/ageing/afae052 PMC10960072 38520141

[14] Hou L, Lei Y, Li X, et al. Effect of protein supplementation combined with resistance training on muscle mass, strength and function in the elderly: a systematic review and meta-analysis. J Nutr Health Aging. 2019;23(5):451–458. doi: 10.1007/s12603-019-1181-231021362

[15] Kirwan RP, Mazidi M, Rodríguez García C, et al. Protein interventions augment the effect of resistance exercise on appendicular lean mass and handgrip strength in older adults: a systematic review and meta-analysis of randomized controlled trials. Am J Clin Nutr. 2022;115(3):897–913. doi: 10.1093/ajcn/nqab355 34673936

[16] Liao CD, Tsauo JY, Wu YT, et al. Effects of protein supplementation combined with resistance exercise on body composition and physical function in older adults: a systematic review and meta-analysis. Am J Clin Nutr. 2017;106(4):1078–1091. doi: 10.3945/ajcn.116.143594 28814401

[17] Witard OC, Bannock L, Tipton KD. Making sense of muscle protein synthesis: a focus on muscle growth during resistance training. Int J Sport Nutr Exerc Metab. 2022;32(1):49–61. doi: 10.1123/ijsnem.2021-0139 34697259

[18] Paoli A, Cerullo G, Bianco A, et al. Not only protein: dietary supplements to optimize the skeletal muscle growth response to resistance training: the current state of knowledge. J Hum Kinet. 2024;91(Spec Issue):225–244. doi: 10.5114/jhk/186660 38689582 PMC11057611

[19] Bhasin S, Travison TG, Manini TM, et al. Sarcopenia definition: the position statements of the sarcopenia definition and outcomes consortium. J Am Geriatr Soc. 2020;68(7):1410–1418. doi: 10.1111/jgs.16372 32150289 PMC12132920

[20] Ahuja JKC, Moshfegh AJ, Holden JM, et al. USDA food and nutrient databases provide the infrastructure for food and nutrition research, policy, and practice. J Nutr. 2013;143(2):241s–249s. doi: 10.3945/jn.112.170043 23269654

[21] von Elm E, Altman DG, Egger M, et al. The strengthening the reporting of observational studies in epidemiology (STROBE) statement: guidelines for reporting observational studies. Lancet. 2007;370(9596):1453–1457. doi: 10.1016/S0140-6736(07)61602-X 18064739

[22] Hasnain SR, Buendia JG, Bradlee ML, et al. Dietary protein, skeletal muscle mass, and obesity risk in adolescent girls. FASEB J. 2012;26(S1):.1011.1011–.1011.1011. doi: 10.1096/fasebj.26.1_supplement.1011.11

[23] Mott MM, Singer MR, Bradlee ML, et al. Protein intake is associated with lower body fat and higher skeletal muscle mass in late adolescence. FASEB J. 2017;31(S1):.29.27–.29.27. doi: 10.1096/fasebj.31.1_supplement.29.727682203

[24] Volterman KA, Moore DR, Breithaupt P, et al. Timing and pattern of postexercise protein ingestion affects whole-body protein balance in healthy children: a randomized trial. Appl Physiol Nutr Metab. 2017;42(11):1142–1148. doi: 10.1139/apnm-2017-0185 28683243

[25] Karagounis LG, Volterman KA, Breuillé D, et al. Protein intake at breakfast promotes a positive whole-body protein balance in a dose-response manner in healthy children: a randomized trial. J Nutr. 2018;148(5):729–737. doi: 10.1093/jn/nxy026 30053279

[26] Damayanti R, Wiratama Natsir MP, Annisa I, et al. Protein intake and number of children associated with nutritional status. J Pak Med Assoc. 2021;71(Suppl 2):S99–s102. 33785951

[27] Lind MV, Larnkjær A, Mølgaard C, et al. Dietary protein intake and quality in early life: impact on growth and obesity. Curr Opin Clin Nutr Metab Care. 2017;20(1):71–76. doi: 10.1097/MCO.0000000000000338 27749711

[28] Wu G. Dietary protein intake and human health. Food Funct. 2016;7(3):1251–1265. doi: 10.1039/C5FO01530H 26797090

[29] Garcia-Iborra M, Castanys-Munoz E, Oliveros E, et al. Optimal protein intake in healthy children and adolescents: evaluating current evidence. Nutrients. 2023;15(7):1683. doi: 10.3390/nu15071683 37049523 PMC10097334

[30] Cruz-Jentoft AJ, Bahat G, Bauer J, et al. Sarcopenia: revised European consensus on definition and diagnosis. Age Ageing. 2019;48(4):601. doi: 10.1093/ageing/afz046 PMC6593317 31081853

[31] Celis-Morales CA, Petermann F, Steell L, et al. Associations of dietary protein intake with fat-free mass and grip strength: a cross-sectional study in 146,816 UK biobank participants. Am J Epidemiol. 2018;187(11):2405–2414. doi: 10.1093/aje/kwy134 29961893

[32] Majid HA, Ng AK, Hairi NN, et al. SUN-PO257: dietary protein intake and hand grip strength among adolescents: finding from Malaysian health and adolescents longitudinal research team study (MyHeART). Clin Nutr. 2019;38:S154. doi: 10.1016/S0261-5614(19)32887-0 PMC6597745 31248920

[33] Jun S, Cowan AE, Dwyer JT, et al. Dietary protein intake is positively associated with appendicular lean mass and handgrip strength among middle-aged US adults. J Nutr. 2021;151(12):3755–3763. doi: 10.1093/jn/nxab288 34494110 PMC8826630

[34] McLean RR, Mangano KM, Hannan MT, et al. Dietary protein intake is protective against loss of grip strength among older adults in the Framingham offspring cohort. J Gerontol A Biol Sci Med Sci. 2016;71(3):356–361. doi: 10.1093/gerona/glv184 26525088 PMC5864162

[35] Ng AK, Hairi NN, Dahlui M, et al. The longitudinal relationship between dietary intake, physical activity and muscle strength among adolescents. Br J Nutr. 2020;124(11):1207–1218. doi: 10.1017/S0007114520002202 32624008

[36] Koletzko B, Demmelmair H, Grote V, et al. High protein intake in young children and increased weight gain and obesity risk. Am J Clin Nutr. 2016;103(2):303–304. doi: 10.3945/ajcn.115.128009 26791192

[37] Hörnell A, Lagström H, Lande B et al. Protein intake from 0 to 18 years of age and its relation to health: a systematic literature review for the 5th Nordic Nutrition Recommendations[J]. Food Nutr Res. 2013;57. 10.3402/fnr.v57i0.21083 PMC3664059 23717219

[38] Rodriguez NR. Optimal quantity and composition of protein for growing children. J Am Coll Nutr. 2005;24(2):150s–154s. doi: 10.1080/07315724.2005.10719457 15798083

[39] Tipton KD. Gender differences in protein metabolism. Curr Opin Clin Nutr Metab Care. 2001;4(6):493–498. doi: 10.1097/00075197-200111000-00005 11706282

[40] Arabi A, Tamim H, Nabulsi M, et al. Sex differences in the effect of body-composition variables on bone mass in healthy children and adolescents. Am J Clin Nutr. 2004;80(5):1428–1435. doi: 10.1093/ajcn/80.5.1428 15531697

[41] Burd NA, Tang JE, Moore DR, et al. Exercise training and protein metabolism: influences of contraction, protein intake, and sex-based differences. J Appl Physiol. 2009;106(5):1692–1701. doi: 10.1152/japplphysiol.91351.2008 19036897

[42] Larson KR, Russo KA, Fang Y, et al. Sex differences in the hormonal and metabolic response to dietary protein dilution. Endocrinol. 2017;158(10):3477–3487. doi: 10.1210/en.2017-00331 28938440

[43] Zhang Q, Wang Y. Socioeconomic and racial/ethnic disparity in Americans’ adherence to federal dietary recommendations. J Acad Nutr Diet. 2012;112(5):614–616. doi: 10.1016/j.jand.2012.02.008 22709764 PMC3378984

